# Can wrist-worn devices and a smartphone application influence arm activity in children with unilateral cerebral palsy? A proof-of-concept study

**DOI:** 10.3389/fresc.2022.1060191

**Published:** 2023-01-30

**Authors:** Amie Turner, Dan Jackson, Eleanor Officer, Chelsy Boyne-Nelson, Zosia Zielinska, Divya Dinraj, Jessica Blickwedel, Tom Nappey, Tim Rapley, Heather Turpin, Jill Cadwgan, Janice Elizabeth Pearse, Anna Purna Basu

**Affiliations:** ^1^ School of Psychology, Newcastle University, Newcastle Upon Tyne, United Kingdom; ^2^ Open Lab, School of Computing, Newcastle University, Newcastle Upon Tyne, United Kingdom; ^3^ Newcastle University School of Biomedical, Nutritional and Sport Science, Newcastle Upon Tyne, United Kingdom; ^4^ Northern Foundation School, Newcastle Upon Tyne, United Kingdom; ^5^ National Innovation Centre for Ageing, The Catalyst, Newcastle Upon Tyne, United Kingdom; ^6^ Department of Social Work, Education and Community Wellbeing, Northumbria University, Newcastle Upon Tyne, United Kingdom; ^7^ Evelina London Children’s Hospital, Guy’s and St Thomas’ NHS Foundation Trust, London, United Kingdom; ^8^ Population Health Sciences Institute, Newcastle University, Newcastle Upon Tyne, United Kingdom; ^9^ Therapy Services, Newcastle Upon Tyne Hospitals NHS Foundation Trust, Newcastle Upon Tyne, United Kingdom; ^10^ Paediatric Neurology, Great North Childrens Hospital, Newcastle Upon Tyne, United Kingdom

**Keywords:** unilateral cerebral palsy, upper limb, therapy, child, intervention, wrist-worn device, smartphone application

## Abstract

**Aim:**

To determine whether a wrist-worn triaxial accelerometer-based device and software (including smartphone application), incorporating feedback, is feasible, acceptable, and can lead to increased affected upper limb use during everyday activities in children with unilateral cerebral palsy (UCP).

**Methods:**

***Study design*:** Mixed methods proof of concept study. ***Participants:*** Children aged 8–18 years with UCP; age-matched typically developing controls (“Buddies”), therapists. ***Intervention:*** *Baseline (2 weeks):* devices recorded arm activity. *Active feedback (6 weeks):* devices also gave vibratory prompts if affected arm activity fell below pre-set personalised thresholds (UCP group only; control group continued as per *Baseline*). *Final 2 weeks:* as baseline. Both groups accessed a smartphone application providing feedback on relative arm motion throughout the study. ***Assessment and analysis:*** ABILHAND-Kids questionnaires and MACS classifications captured baseline participant characteristics (UCP group). Accelerometer data was used to calculate relative arm activity (signal vector magnitude) corrected for time worn/day, and trends in relative arm activity examined using single case experimental design (both groups). In-depth interviews with families, “Buddies” and therapists assessed feasibility and acceptability of implementation. A framework approach was used for qualitative data analysis.

**Results:**

We recruited 19 participants with UCP; 19 buddies; and 7 therapists. Five participants (two with UCP) did not complete the study. Baseline mean (stdev) ABILHAND-Kids score of children with UCP who completed the study was 65.7 (16.2); modal MACS score was II.

Qualitative analysis demonstrated acceptability and feasibility of the approach. Active therapist input for this group was minimal. Therapists appreciated the potential for summary patient data to inform management. Arm activity in children with UCP increased in the hour following a prompt (mean effect size *z* = 0.261) for the non-dominant hand, and the dominant hand (*z* = 0.247). However, a significant increase in affected arm activity between baseline and intervention periods was not demonstrated.

**Discussion:**

Children with UCP were prepared to wear the wristband devices for prolonged periods. Whilst arm activity increased bilaterally in the hour following a prompt, increases were not sustained. Delivery of the study during the COVID-19 pandemic may have negatively influenced findings. Technological challenges occurred but could be overcome. Future testing should incorporate structured therapy input.

## 1. Introduction

Cerebral palsy (CP) is the most common motor disorder of childhood (1). Unilateral (hemiplegic) CP is the most frequent form of CP (2). The effect of the condition on hand and arm function on one side of the body can impact on activities of daily living (2, 3), quality of life (4), employment (5) and independence (6).

Effective interventions for upper limb function in children/young people with UCP include constraint-induced movement therapy (7–9) and hand-arm bimanual intensive therapy (10). However, these processes are highly resource- and time- intensive, requiring 1 : 1 therapist support for many hours to achieve improvement. In practice, therapy input for the upper limb for many children is more limited. A typical example (based on the experience of clinical members of the authorship group and feedback from parents) might be a review by a community occupational therapist once a term at school followed by provision of advice to the family and staff. Many children in the UK with UCP are reviewed then discharged by therapy services and therefore have long periods during which they receive no therapy at all.

As paying for private therapy provision is not a realistic option for most families, the burden of supporting their child's use of their affected arm often falls to the parents themselves. This is a challenge for several reasons. Firstly, children with CP require even more practice than typically developing children in order to learn new motor skills (11), so there is a significant time investment. Secondly, parents can struggle with the amount of prompting required to motivate their child to stay engaged with relevant tasks (Brown et al., under submission).

Technology is increasingly used to support clinical interventions (12), monitor activity levels (13), support behaviour change (14) and encourage self-management for medical conditions (15, 16). For adults with stroke, a wrist-worn device was developed which could monitor arm activity using accelerometry and emit a prompt when movement had fallen beyond an agreed threshold in the preceding hour. In a pilot trial this led to a 16% increase in arm activity in the hour after a prompt (17).

Children are not “little adults”, and a study design which works for adults with UCP following stroke requires modifications for optimal use in children. This work builds on previous studies (17) by recording from both wrists, so as to determine the baseline difference in activity between sides and control for within-person variability in overall level of activity, which might be expected to be higher in children than in adults. Furthermore, a smartphone application was developed, with a game for children to interact with as a reward for daily compliance with wristband wearing. Finally, each child in the study had a typically developing age-matched “buddy” who also took part but did not receive prompts to move. The aim of including the buddy was to provide peer support as well as to provide further normative data on relative limb movements in this age group. These decisions regarding study design were discussed in focus groups with stakeholders prior to undertaking the current study (Brown et al., accepted). The updated MRC complex intervention framework was used in designing the study (18).

This proof-of-concept study was an adaptation of the WAVES study (17) for children. Individuals personally managed their improvements in arm movement by responding to feedback from wrist worn devices and a smartphone application. This approach is a promising real-world solution for children with UCP, empowering them to take control of their own improvements using technology with which they are familiar and comfortable.

This study aimed to determine: acceptability of the wrist worn devices and the related smartphone application in different environments; the feasibility of the protocol in terms of individual commitment for the duration of the study; and the degree to which technological challenges could be overcome. Finally, the study aimed demonstrate proof of concept that this approach can increase activity of the affected arm in children with UCP.

## 2. Methods

The study used a mixed methods (concurrent triangulation) design. Thus, both qualitative and quantitative data were collected through the study, and final interpretation relied on integration of the findings. For example, information on commitment over the duration of the study could be obtained by studying wear time of the devices over the 10-week period and from data collected during telephone calls and interviews.

### 2.1. Participants

#### 2.1.1. Inclusion criteria

Participants included children and young people with UCP; their “buddies”; parents and therapists.

Children and young people with UCP included in the study were between 8 and 18 years old and had a Manual Ability Classification (MACS) level I–III (19). Each child/young person with UCP selected a “buddy” who was a typically developing age-matched peer. Buddy systems can encourage children to engage in physical activity (20), and can foster inclusiveness and mutual support (21).

Fully informed written consent (parents and those aged 16 and above) was required, with additional assent from participating children. With consent from the parents of the children/young people with UCP, their therapists (physiotherapists and/or occupational therapists) were asked to take part in an in-depth interview. Therapists were eligible if their involvement with the child involved providing input related to upper limb function. Due to constraints on interpretation service provision, adequate command of the English language was also an inclusion criterion.

#### 2.1.2. Exclusion criteria

Children who were registered blind or partially sighted, and those with significant cognitive and/or language deficits were excluded from participation, as they would likely struggle to make use of the smartphone application. A simple test for ability to detect a vibratory cue to the affected wrist (which constituted the prompts provided in the study) was undertaken; inability to detect the cue was a criterion for exclusion. Involvement in another research study likely to interfere with the conduct of the current study was also an exclusion criterion.

#### 2.1.3. Identification and recruitment

Children/young people with UCP who were eligible to take part in the study were identified by clinicians at the Newcastle upon Tyne Hospitals NHS Foundation Trust, and by clinicians at the Evelina Children's Hospital in London. These children/young people were then referred through their regional clinics once a member of the team discussed the research project with them. Additionally, the study was advertised through local self-help groups for families with children/young people with UCP (former HemiHelp groups) in Newcastle and London.

Once referred, the families and children/young people were given more information about the study and were provided with an age-appropriate information sheet. They then considered their participation in the study and at any point were able to ask a member of the team any questions. Those who consented to participate were given a flyer to provide to the buddy and their family. The potential buddy's family could then discuss the study in detail with a researcher if interested and decide on their participation. All participants were made aware they could discontinue participation at any time without providing a reason, even after signing a consent form. Ethical approval for the study was provided by West Midlands – Edgbaston Research Ethics Committee, reference 19/WM/0257.

### 2.2. Sample size

The recruitment target was 20 children/young people with UCP and 20 typically developing “buddies”. This sample size was expected to be sufficient for qualitative data code saturation (22), permitting a range of scenarios in regards to demographics across two sites and functional ability to illustrate proof of concept. Recruitment started in January 2020, was delayed due to the COVID-19 pandemic, and data collection was complete by end September 2021.

### 2.3. Baseline assessments

Baseline assessments were undertaken face to face where possible though due to the COVID-19 pandemic, some were by necessity undertaken remotely.

#### 2.3.1. Manual ability classification system

The Manual Ability Classification System (MACS) (19) is a validated 5-point classification system describing how those with cerebral palsy use their hands in everyday activities. MACS I represents the highest level of self-initiated hand use. Parents of children with UCP, or these children/young people themselves, completed the assessment by following the simple questions on the flow chart. The MACS is stable over time: once a child has been assigned a MACS classification, this is unlikely to change.

#### 2.3.2. ABILHAND-Kids questionnaire

The ABILHAND-Kids questionnaire was used to gauge manual ability whilst performing daily tasks, in children with UCP (23). The questionnaire was completed by the parents (taking around 5 min) as per standard practice and was based upon their perception of their child's manual ability in undertaking various specified tasks, regardless of which hand is used. The assessment is validated and is suitable for children aged 6–15 years. Each item is rated on a 3-point scale as to whether the child would find that task impossible, difficult or easy. The questionnaire scoring system is Rasch-based and is completed online. For those aged 16 years and over, the ABILHAND questionnaire (self-completed) was used instead.

Two other assessments were planned for the study but could not be undertaken for most participants because of the requirement to minimise face to face contact during the COVID-19 pandemic. The Assisting Hand Assessment (AHA) or equivalent Adolescent-AHA for those age 13 years and above (24, 25) was planned to be undertaken for children with UCP at baseline, as a measure of performance of the affected hand in bimanual tasks. This was only possible for 7 participants. The Tyneside pegboard test was intended to be undertaken by all children and young people in the study (26), to assess unimanual and bimanual dexterity, but again was not usually completed due to the requirement or parental preference for the assessments to be undertaken virtually.

### 2.4. Equipment

#### 2.4.1. Wrist-worn devices

**Figure 1:**
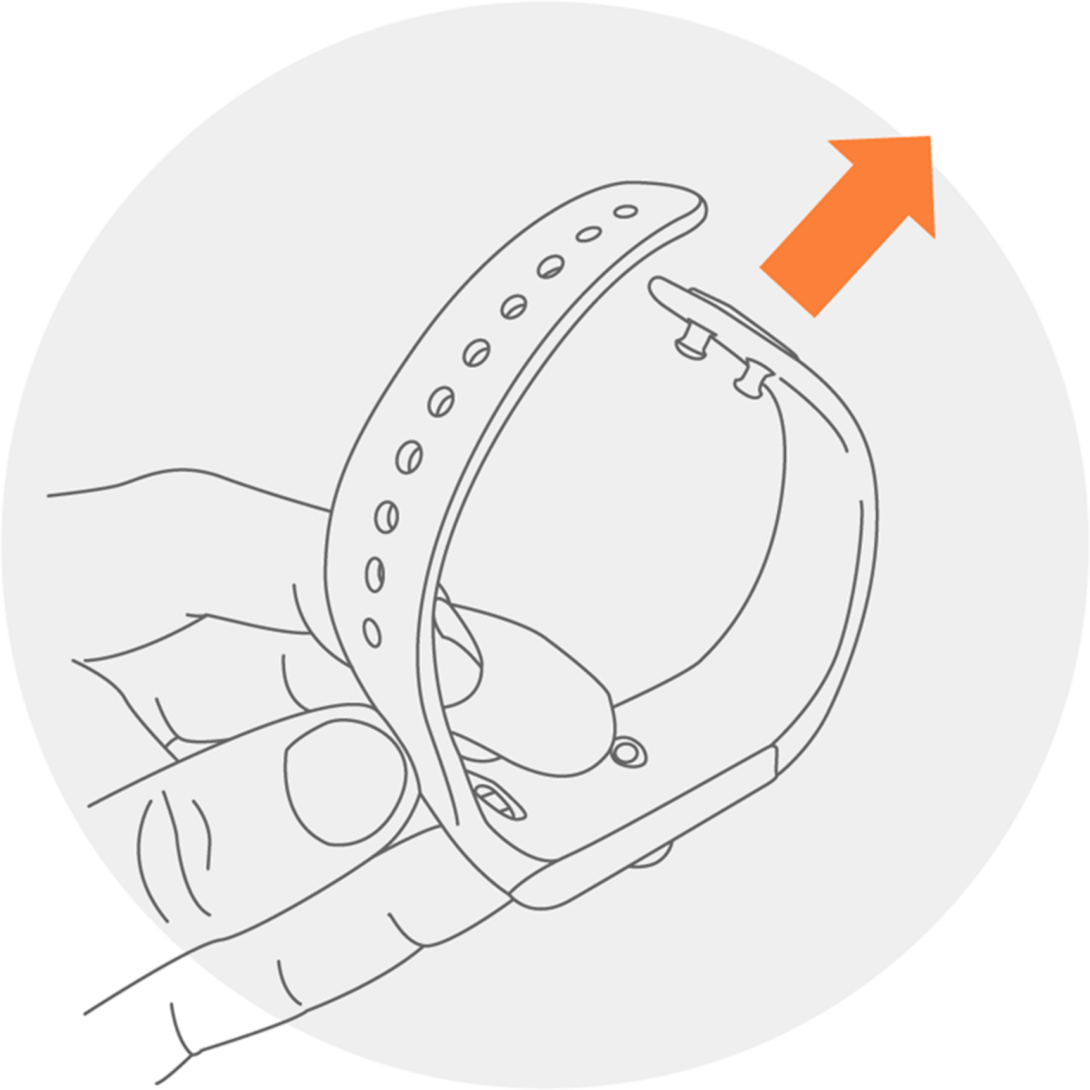
Wristband clasp mechanism.

Children/young people in the study were each issued with two AxLE bands. The AxLE band was developed as part of Newcastle University's Open Movement project Open Movement (digitalinteraction.github.io), and consists of a low-cost wrist-worn fitness band (iWown i5 Plus device) reprogrammed with open source firmware developed for the project and further customised for this study. The device hardware contains a Bluetooth enabled microcontroller and triaxial accelerometer sensor. The sensor data was converted to summary data and stored on the device. The choice of device was influenced by prior focus groups (Brown et al., accepted) in which the device size in relation to children's wrists, ease and security of fastening and unfastening the device, and comfort, were explored. The lightweight silicone bands were also chosen to have a low risk of skin reaction. The chosen devices also had to have enough onboard memory to cater for at least an 8-hour window (school day) without relying on data transmission to the server during this time. The recording device slotted into the front of the wristbands and was removable for charging (Figure 1). It had not been possible to find appropriate wristbands designed to fit young children.

The devices also had a vibratory output which was used as a signal to prompt increased movement of the affected arm in children with UCP where this fell below a pre-set threshold (based on the average activity in the baseline period). The device interface i.e., the screen on the wristband (which was activated by tapping it lightly) displayed the date and time, showed to what extent the battery was charged (with battery life around 7 days), and indicated L or R to help with correct allocation to each wrist. Devices could also be sent into “shipping mode” for delivery or return by post if required. Participants were also issued with a dual USB charger so that both devices could be charged at once at home.

#### 2.4.2. Smartphone application

A mobile application on the participants' smartphone automatically retrieved the wristband data wirelessly *via* Bluetooth and sent it to our server for further analysis. This application was produced and made available through the Google Play store for the duration of the study. It was also downloaded on to Android phones which were supplied to children who did not have access to an Android phone or tablet of the appropriate specification. The limitation to Android compatibility was due to time and resource constraints on the project. The wristbands had to be “synchronised” with the application each day to download new data from the bands to the phone, upload this data to the project server, and update the bands' configuration (including synchronizing their internal clocks). The application dashboard also indicated the remaining battery life for the wristbands, and when synchronisation had last occurred. A separate tab allowed for display of activity of each arm per day, and a more detailed view of hourly activity was available for any one day. This included the percentage of time between 8 am and 8 pm that the devices were estimated to have been worn on any one day (based on prior work determining the threshold for activity recorded during periods of non-wear, described in more detail in “Quantitative Data Cleaning”); the “balance” (an indication of the relative amount of activity from the affected vs. the unaffected side, based on the difference between sides divided by the sum of activity on both sides); the number of prompts to move received that day (for those with UCP), and an overall score out of 10 derived from these results. Relative activity of each arm for each day was visible to participants *via* the application, presented as a violin plot broken down into one-hour bins.

For buddies, the score was simply based on the fraction of the 12 h that the devices were assessed as having been worn; for children with UCP, this was moderated by the “balance”, so that increase in relative movement of the affected arm could lead to an increase in score. This score was used to help “earn” points to use in playing a simple game on the app each day as a reward: this feature was added following recommendations from focus groups undertaken in the participatory design phase of the study.

The game chosen needed to be simple to use and fun. Implementation was inspired by casual “launcher” genre games such as the “Learn to Fly” series and used the TwoCan project logo to develop a toucan character. “Points” earned (co-operatively with the buddy) could be used to achieve “upgrades” to achieve higher ranks in the game, awarded for different aspects of performance such as duration of flight, maximum altitude, airspeed etc. The aim was for the game to provide sufficient variety and interest to motivate ongoing use over a 10-week period. It was designed for single handed play and to run well on mobile devices, with touch screen controls and short sessions suitable for opportunistic play.

### 2.5. Instructions and internet safety

Participants were provided with information about safe internet use as part of the project. This included the Safer Internet Centre (www.saferinternet.org.uk/advice-centre/young-people/resources-11-19s) and the Online Safety Guide from Internet Matters: https://www.internetmatters.org/advice/online-safety-guide. They were also provided with instructions regarding the use of all equipment provided including access to the TwoCan website The TwoCan Project - TwoCan, and contact details of the study team in case of difficulties arising between scheduled contact calls.

### 2.6. Intervention

The children/young people were asked to wear the wristbands from 8am to 8pm for 10 weeks. They were removed at night, for charging, and for activities which might be detrimental to the devices such as swimming, washing etc. During the first 2 weeks, for both groups the devices recorded arm activity as a baseline but did not provide any prompts to encourage movement. Personalised thresholds for activity of the affected arm were then set for children with UCP, based on the baseline data. More specifically, the average activity of the affected upper limb during each hour between 8 am to 8 pm from the baseline period was used to set the prompt threshold. Thus, the starting requirement was for the children to continue moving their affected upper limb on average as much as they did during the same time of day during the baseline period. During the next 6 weeks a brief vibration through the wristband on the affected wrist was sent if the mean activity on that side had not reached the set prompt threshold in the previous hour. The prompt frequency was capped at 1 h based on prior feedback that more frequent prompts would be potentially intrusive. The children and families were aware that the vibration was a prompt to increase activity of the affected side. It was possible for a researcher to adjust the thresholds for the prompts, through a web-based login, if necessary. These changes were based on the previous week's activity and using a change of 5%. For example, if a child had required very few prompts, the threshold could be increased by 5% if the child and family agreed to this. Children/young people were encouraged to view the smartphone application to gain information regarding their progress. Buddies did not receive any prompts but still had access to the graphical information and the game. During the final 2 weeks of the study, children/young people were not given prompts: this was done to see if any change in level of activity was sustained. Children/young people and families had weekly telephone contact with a member of the research team to troubleshoot and obtain feedback.

### 2.7. Outcome measures

#### 2.7.1. Qualitative data

Throughout the study the children/young people and their buddies (and parents of both as appropriate) took part in a weekly telephone call with a member of the research team. The aim was to gather information on acceptability of the device and the application and for troubleshooting. The families were asked about the effect of using the device on social contact, activities and participation, and if the children suffered from any discomfort/fatigue. They were also asked about any ongoing input from any therapist supporting their child's upper limb function. The children/young people were also encouraged to provide feedback. The sessions were audio recorded and transcribed (anonymised) prior to subsequent analysis. Telephone interviews with therapists of participants with UCP were undertaken at the end of the data collection period where their views were explored on the approach. This was because the approach could be of interest to therapists, who might be able to use data from the smartphone application to guide their interventions. Furthermore, we wanted to understand whether therapists had any reservations or thoughts about the approach which would need to be taken into account prior to considering a subsequent large scale evaluative study.

#### 2.7.2. Accelerometry data

Triaxial accelerometers within the wrist worn devices provided minute by minute information in relation to arm activity during the 10-week period. Specifically, they provided the one-minute epoch mean of the absolute value of the acceleration vector's ENMO (Euclidean Norm Minus One), where the Euclidean Norm is band-pass frequency filtered; units of 2^−12^ *g*, where *g* is approximately 9.81 m/s^2^.

Data was originally sampled at 100 Hz from the triaxial accelerometers (“STMicroelectronics LIS3DH”) with orthogonal axes *X*, *Y* and *Z* each measuring 16 bits per axis sample, giving 2^16^ possible values; “twos complement signed value” thus giving a raw value range −32,768 to 32,767. Sensitivity range was set to ±8 *g*, giving the ENMO units indicated above. The ENMO value was calculated per full sample by first taking the scalar vector magnitude then subtracting 1 *g*. The ENMO value was filtered with a Butterworth bandpass IIR filter configured for 100 Hz input and using the nominal cut-off frequencies of 1 Hz and 20 Hz and 16 bit quantization as used in a previous study (17). The resulting frequency filtered ENMO absolute value was accumulated into a total for the 60 s epoch. Each epoch mean was calculated and stored.

To trigger prompts, a “window” was maintained for the most recent summary movement values over the previous 60 min. The rectangular filtered output of this window was compared with the currently active prompt threshold (if set): if below the threshold, and if at least 60 min had elapsed since the previous prompt, then a vibrating prompt was given to the affected limb. The number and timing of prompts was recorded.

### 2.8. Data analysis

#### 2.8.1. Qualitative data analysis

Qualitative analysis was conducted through a framework analysis approach, following standard procedures (23). This included open and focused coding, constant comparisons and memoing (24), deviant case analysis (25), and mapping (26). The data were then aligned to the Person-Environment-Occupation (PEO) Model of occupational performance (27). This was because a key element was to understand how the components of the intervention interacted and how the context may influence these components. The PEO model is used by Occupational Therapists, and recognises that performance is dependent on the dynamic relationship between the individual, the role, and the environment they are in.

#### 2.8.2. Quantitative data cleaning

Wristband data collected between 8 am and 8 pm between the start and finish date for each participant was considered for analysis. First, estimated non-wear periods were excluded. The cut-off for non-wear was determined based on prior analysis of minute-by-minute activity data from 12 am to 6 am for 5 nights from 5 participants who were not wearing the devices at those times. To do this, standard deviations of activity were calculated for 15-minute rolling window intervals, to identify the highest standard deviation for the non-wear period. The empirical cut-off identified was a standard deviation of 0.77 units. If activity for either arm fell below this cut-off, data for both sides was withdrawn for the period of concern. Further data cleaning involved checking the logged phone call data for each participant and ensuring data exclusion on days when bands were not worn; worn on the wrong wrists; or lost. Data were removed on days where the wristbands appeared to have been worn for less than 360 min, as this was less than half of the required 720 min of wear per day. Finally, visual inspection of the ratio of non-dominant to dominant arm activity was undertaken, looking for evidence of days where the bands were likely on the wrong wrists (i.e., the ratio was approximately the reciprocal of the usual ratio for that participant); and we excluded those days from analysis. The mean activity of each arm was then calculated for each hour of each day, for each participant, as was the total time each day during which the participant appeared to have been wearing both devices.

#### 2.8.3. Arm activity analysis: single case experimental design

Once cleaned, the data was analysed using a Single Case Experimental A-B Design Approach (28). This approach tests the effect of an intervention on a case-by-case basis and allows analysis to account for the differences in variables for each participant over time. For each participant a time series graph allowed visual inspection of the daily activity ratio (non-dominant/dominant hand, corrected for time worn). Each graph plotted a median line through the baseline (*A*) phase data, extended through the intervention (*B*) phase (29). The percentage of data points in the intervention period exceeding the median for the baseline period (PEM) was then calculated. Cases with PEM above 50% suggested a possible improvement in the intervention phase and were analysed further using the Tau *A* vs. *B* test (equivalent to Mann–Whitney *U*) (30). The website Visual aids & Nonoverlap indices (shinyapps.io) was used for both graphical depiction and quantitative analysis.

#### 2.8.4. Analysis of prompts

For participants with UCP, the mean number of prompts per day during the intervention period was calculated and compared between the first vs. second half of that period.

It was important to determine whether there was a change in arm activity just after a prompt compared with just before a prompt. This was done for both dominant and non-dominant arms. To prevent contamination of the activity data by the vibration caused by the prompt, the minute during which the prompt was given was excluded. The effects of the prompt were compared on two timescales: 5 min before vs. 5 min after, and 60 min before vs. 60 min after. Analysis was undertaken on a case-by-case basis using the Wilcoxon signed rank test due to the large inter-individual variation in prompt number and nonparametric nature of the data. IBM SPSS Statistics (version 25.0, Armonk, NY: IBM Corp.) was used for statistical analysis.

## 3. Results

### 3.1. Participant characteristics

**Figure 2:**
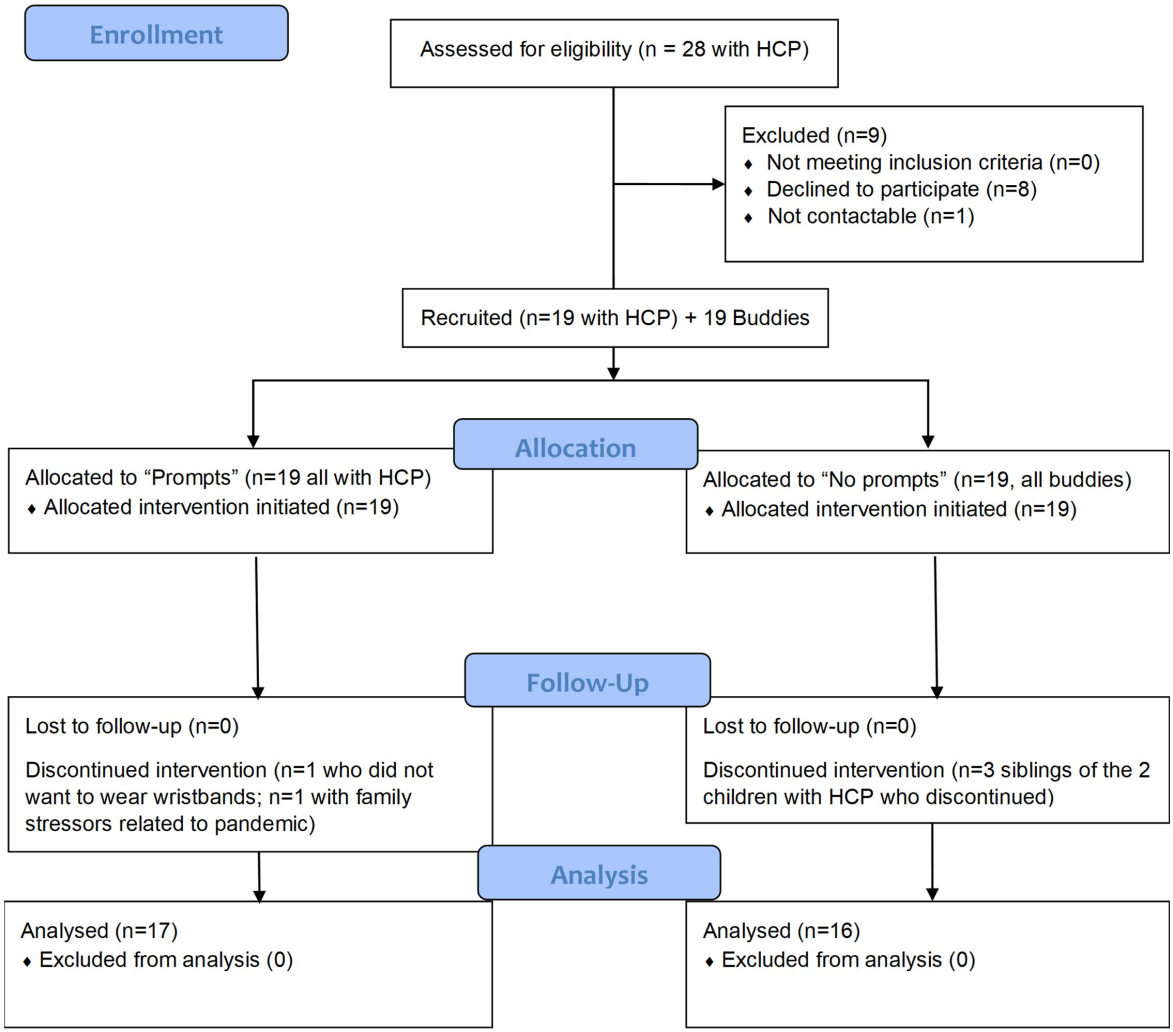
Participant flow diagram.

Figure 2 shows the participant flow diagram. Nine families with a child with UCP who were approached declined to participate. Reasons given were that the child disliked having anything on their wrist (*n* = 2); coping with the equipment when living across two households; the child declined to participate; imminent (*n* = 1) or recent (*n* = 1) vagal nerve stimulator implantation; the child undertook daily swimming; and concerns about demands on the child's time. One family did not respond to follow up calls after having demonstrated initial interest.

Nineteen children with UCP and 19 buddies took part in the study. Most participants with UCP were able to identify a buddy themselves but for 2 participants the research team identified a buddy on behalf of the participant. Five participants (2 with UCP and their 3 siblings) discontinued involvement in the study prior to completion, in one case because the young person with UCP did not wish to wear the wristbands, and in the other case due to pandemic-related stressors on the family. Thus, data was collected and analysed from 33 children and their families.

Fifteen children were seeing a physiotherapist, 9 were seeing an occupational therapist and 8 were having some form of upper limb therapy. From the Newcastle site, 4 physiotherapists were interviewed, each of whom was supporting a different participant in the study. From the London site, 2 occupational therapists were interviewed, one of whom was supporting two different participants, and one physiotherapist was interviewed, who was supporting two study participants. In total 7 therapists were interviewed, in relation to 7 participants with UCP.

Only 12 families owned an Android phone; phones were loaned to the remaining families for the duration of the project.

Table 1 summarises the participant characteristics of those whose data was included in the analysis.

**Table 1:** Characteristics of participants included in the analysis. The two children with UCP who did not complete the study were aged 9 and 11 years, with MACS of 1 and 3 respectively and were both male.

	UCP	Buddies
Number	17	16
Median Age (years) (IQR) NB overall range 8–17	10 (3)	11 (5)
Number of males	8	7
Number with right hand dominance	8	13
MACS level		n/a
I	1	
II	12	
III	4	
ABILHAND-Kids questionnaire percentage score Mean (stdev.)	65.7 (16.2)	n/a
Wrist circumference (cm), range	12.0–17.9	13.2–17.0
Difference in wrist circumference between wrists (cm): mean (stdev.)	1.08 (0.46)	0.14 (0.19) though missing data from 5 participants

### 3.2. Analysis of qualitative interviews: PEO model

Themes identified from the qualitative analysis were a good fit to the Person Environment Occupation model (27), where “Occupation” was interpreted as participation in the study.

#### 3.2.1. Personal factors

Personal factors influenced how participants interacted with the study. This included the age of the child, with a tendency for greater enthusiasm for participation at the younger end of the study age range.

“I think as you kind of move closer towards the teenage bracket, like you say, there's a lot more opting out for lots of things as opposed to opting in.” (T10001, Physiotherapist)

The motivation to take part differed between parents, children with UCP and buddies. Parents were highly motivated to find solutions to improve their child's upper limb function. Some children with UCP, especially the younger ones, found it exciting to wear the devices and were encouraged by the potential to help not only themselves but other children with UCP through research. One child with UCP had a different agenda, which was to improve upper limb appearance rather than function. The child agreed however that prompts given during the study could also remind participants to readjust their upper limb posture.

Children with UCP found it exciting to be wearing wrist worn devices. They liked the idea that it would help them but also help other children with UCP. Similarly, some buddies were motivated by being able to help their friend or sibling.

“I’m helping [name of child with UCP] in a way no one else could.” (200016, Buddy)

Some children felt more comfortable than others with wearing two wristbands. Whilst some saw them as “cool”, others preferred to hide them under a long-sleeved coat and might take them off if they were with their friends. Having a buddy who also wore two bands was seen as helping to normalise the experience for some.

"It made me a bit more … because I knew I was not the only one that was going to have two watches on” (100002, child with UCP)

Participants had a good understanding of why they were doing the study and the confidence to explain this to others, though some felt jaded by having to do so many times. Those who struggled with this could show others a small card summarising the study – this was found to be useful to show to teachers.

#### 3.2.2. Environmental factors

Various environmental factors, both anticipated (school and home settings; therapeutic environment) and unanticipated (COVID-19 pandemic) influenced the intervention.

##### 3.2.2.1. School environment

Schools were in general very supportive of the study and appropriately curious. Families reported that teachers could see that in practice the vibration prompts were relatively quiet and did not distract other students. One teacher had considered using the study as a theme for a class project. Some participants were distracted by prompts or unsure about how to respond when at school:

"Well, I was doing other stuff, because sometimes I was in lessons when I was doing it so then I had to write, but I can't write with my right hand” (100002, child with UCP)

There was some anxiety from participants that it might be difficult to inform all staff members and classmates about their engagement in the study and that this could lead to problems in large secondary school settings.

"You can't just send out 40 letters to all your classmates, explaining what you're wearing. You can’t send out 5 or 6 letters to your different teachers, explaining what you're wearing” (200017, child with UCP)

In practice this was not encountered as a problem, possibly because the “business cards” given to participants could be shown in school to anyone requiring further information about the study.

##### 3.2.2.2. Home environment

One therapist mentioned the likely variability between families in terms of general activity and engagement in therapeutic play. This was borne out by parental comments regarding their role in motivating their children to engage in the study. Parents would often prompt their children to wear the wristbands, play the game, or move their affected arm, which complicates the interpretation of the influence of the wristband-produced prompts.

"I know when George [sibling of child with UCP] did hear them he was prompting her and telling her to move her arm and stuff. So, she was getting, like, double prompts” (100010/11, Mum)

However, some parents noticed they were not prompting their child to move their hand as much, feeling that the wristband prompts would do this for them:

"Kate getting older and getting into, sort of, teenage years and it's something that she can, erm, can take control of. And it's something that she's got without any other influence from other therapists or parents or, you know, nagging people, saying, “Have you done this?”“ (200015, Mother of child with UCP)

Families varied in their degree of comfort with their child having access to a phone to use the TwoCan app including the game. Some parents would look at the data together with their child, but some older participants took full control of the equipment.

##### 3.2.2.3. Therapeutic environment

In general, parents of children with UCP felt their children did not get enough therapy through the NHS. They had to push hard to be seen or for extra support to be put in place, perhaps especially because of the backdrop of the pandemic.

"Most of them just focus on her legs, the physios in [Town] aren't interested above waist generally. That's saved for the OTs, obviously the OTs have disappeared as well, so we haven't, or very little communication. They sent me through the standard, I think it's like a 20-page document of bimanual tasks you can do with kids, but that's all I've had from them really.” (100002, Mother of child with UCP)

There were no reports of the TwoCan approach conflicting with any ongoing therapy. Use of a “second skin” Lycra splint did not prevent children from being able to detect the vibrating prompts, perhaps because of the sound emitted during the vibration.

OT and PTs interviewed welcomed the idea of having the TwoCan project as part of their practice. They felt it had potential for remote monitoring, allowing for fine-tuned adjustments to prescribed interventions.

"But actually, if this could be used as an additional kind of assessment, intervention and evaluation, it allows us to direct our service, in “Are we giving these children enough?” And try and get a little bit more standardised across, you know, even our service or services in the North-East.” (T10004, Physiotherapist)

However, there were some concerns that a complex process would be difficult to implement by therapists especially on an infrequent basis.

##### 3.2.2.4. COVID-19 pandemic

The Covid-19 pandemic affected many aspects of the study including the school environment, the home environment, peer relations and NHS therapy services provision.

Covid-19 led to multiple periods of school and club closures. Participants were generally less active than usual during “lockdown”, and some could see evidence of this in their study data and worried that it could impact study results:

"And it just goes to show this last couple of weeks we've been kind of stuck at home, his graphs are nowhere near what they were before Christmas kind of thing. Yes, so we haven't been as active with the lockdown” (100009, Mother of Buddy)

The reduced interaction with peers also adversely impacted the buddy system:

"Obviously, speaking to [friend of child] as well, he hasn't seen [friend] as much to be able to talk about if they use the game or chatted about using it when they get home or whatever or talked about how it's felt because they haven't spent as much time in school with one another” (100003, Mother of child with UCP)

Face to face NHS therapy provision was also greatly reduced, with some participants reportedly not seeing a healthcare professional for two years or having remote consultations instead. Families and therapists were aware of the reduced ability to assess movement quality remotely, even in the absence of technical challenges. They were frustrated by the adverse impact of the pandemic on therapy service provision for children with UCP. The TwoCan study was seen as being of particular benefit in this setting because of the provision of ongoing input and remote monitoring, even when face to face assessments were not undertaken.

#### 3.2.3. “Occupation” (intervention-related) factors

Intervention-related factors included use of the wristbands and smartphone application for the duration of the study, the involvement of a “buddy”, ongoing contact with the research team through weekly phone calls, and perception of potential benefit.

There was some apprehension at the start of the study regarding the 10-week duration and the requirement to record data for an inflexible 12-hour period.

"I think, erm, for, erm, if, if- as we're being recorded, I think it should be customised, so Adam gets up at 6:00 every morning so you'd miss two hours of him first thing in the morning but then he will get into the bath about 7:00. So, he needed to take it off an hour earlier. So 8:00 till 8:00 doesn't work for Adam. 6:00 till 7:00 would have worked for Adam.” (100001, Mum of UCP Child)

This apprehension proved to be justified based on quantitative data demonstrating wear time as discussed below.

##### 3.2.3.1. Wristbands

Participants were in general happy with the appearance and comfort of the wristbands though a few reported finding them less comfortable in warmer weather due to sweating. Some participants found the dual clasp system less secure than planned; or the device would detach from the wristband:

“Because he has got quite thin wrists, we had to put them on the tightest setting. So, because of that, because the actual device is quite wide, they spring out of the wristband quite a lot.” [100020/21, Father of child with UCP (20)]

Part way through the study, small bands to help secure the strap in place were introduced, which were found to be helpful. However, it remained the case that most children needed help to put the wristbands on, as anticipated.

“I think with the left hand it would have to be elasticated or something, because obviously she just hasn’t got the fine motor in her right hand to put it on.” (100002, Mother of child with UCP)

The device screen was activated by gently touching it whilst holding it in the horizontal position. The aim of this was to preserve battery life, but some participants and parents found it tricky to activate the screen. Once activated, the screen showed the date, time, battery life and indicated on which wrist the device should be worn. Some participants would have liked additional functionality, but this would have drained the battery quickly. (A battery life of around 1 week was anticipated.) Instructions for charging the battery were provided on the TwoCan website and on a leaflet provided at the start. Families varied as to how easy they found the devices to charge.

Prompts to move, generated as a vibration by the wrist-worn devices, worked well when the bands were fully charged and had been synchronised with the smartphone application. Participants with UCP usually felt that they were receiving the right number of prompts or would request an increase in prompts. Some wanted to have more prompts at weekends than weekdays, to reduce the distraction of having prompts during the school day. Parents started to notice their children responding to prompts, by stretching, “wiggling” or waving their affected arm.

“I really liked it when she had the prompts and, erm, because I could see a definite response, you know. It was an instant thing that would have an instant response throughout the day. It didn’t matter where she was, very easy, erm, so, yeah, I would have- for the prompts and everything I would have really liked that to continue to be honest.” (200015, Mother of child with UCP)

Whilst all participants were able to detect the prompts, one commented that it would be useful to be able to increase the strength of the vibration as this might lead to greater efforts to increase arm activity on the part of the participants.

Some participants struggled initially with the change in routine required to integrate the wearing of wristbands into their day:

“I mean during a school day, it's obviously so much easier because you’re in a little more of a regimented routine of getting in, up and out of the house more quickly. And erm, so there have been a few, a few mornings in the holidays where we suddenly thought, “Oh no, they’re not on.” But erm, it's- yeah, it's, it's been quite easy to just integrate it into our- yeah, into the usual morning routine.” (200015, Mother of child with UCP)

##### 3.2.3.2. Smartphone application

Participants used the smartphone application to view their study data. Some families were frustrated by the synchronisation process between the wristbands and smartphone application, which varied in terms of success and speed.

“Sometimes it took a long time and sometimes it was automatic, so I wasn’t sure when or if it was synchronised or not or- a bit confusing.” (200010, Buddy)

The application was designed for Android phones, though it has the potential to be further developed to include use on Apple devices. Families who were loaned an Android phone struggled to remember to keep the phone charged.

“Fine, apart from when the phone would drop into low power mode. I guess, if you're using a phone normally, you'd notice that sort of thing. It was only because it was the only purpose of the device that we didn’t really notice.” (100003, Dad of UCP Child)

Participants generally looked at the data occasionally throughout the week rather than daily as hoped, with parents citing time constraints as the reason. Their children might have had fewer time constraints, but parents had reservations about their children having access to the phones due to concerns about overall screen time. Most participants found the data easy to understand and interesting.

"When I was doing things like sports, and then it went like super-active, and afterwards I'd take them off to take a shower and then there was nothing. I found that quite funny.” (200,020, Child Buddy)

Parents often showed interest in the data. They appreciated being able to see the difference between their child's affected and unaffected arm activity. Many were pleasantly surprised to see how much their child used their affected arm.

The smartphone application was also used by the children to access a short game as “reward”, during which they could use points earned from study participation and (for those with UCP) arm use. Some parents restricted phone use by their child, affecting access to the game:

“Well, my mummy and daddy won't let me play it." (200009, child with UCP)

One child with UCP struggled to play the game due to difficulty holding the phone with one hand whilst using the other to tap the screen to engage with the game. The rules of the game were quickly understood by most, and the gaming instructions on the TwoCan website were felt to be helpful; however, the game was felt by some to be a bit simplistic with little incentive to continue to engage with it. Others were motivated by the need to earn points:

"It was good because you didn't just get points for free. You actually had to get more active. You're not just going to press on something and they're going to give you loads of points, you have to work for the points” (100009, Buddy)

Overall, participants liked the idea of a game. The challenge was that there was no consensus about what type of game would be optimal for all.

##### 3.2.3.3. Buddy

Some parents of children with UCP were anxious about finding a buddy, and one therapist wondered whether this was due to the personal nature of taking part in an intervention related to a medical condition but in practice the process worked well.

"I sent a group message saying, “[name of child] is taking part in this project, this is what it's about, if you'd be interested in letting your child participate, it will involve X, Y and Z, can you get in touch with me?” I braced myself praying that somebody would be willing and, actually, we got inundated with messages from parents.” (100003, Mum of UCP Child).

The involvement of a buddy was helpful for some children with UCP:

"It felt like I was more motivated because I had someone I could really relate to with it.” (100001, UCP Child)

##### 3.2.3.4. Phone calls

Weekly phone calls were usually undertaken with the parents, with occasional input from the child. The calls were particularly helpful for troubleshooting but if all was going well, parents felt they did not have a lot to add during the phone calls.

#### 3.2.4. Overall perceptions of the intervention

Participants generally said they would recommend this intervention and enjoyed taking part.

"….it made me feel more, kind of like I'm a strong person, I, I can do anything” (200007, child with UCP)

"Yeah. I think, I think they did see value in it. They liked that, you know, it, it looks quite normal, it's quite socially acceptable, and it's providing prompts. And they liked - they really liked the concept of it.” (T200001, OT)

Some families felt that viewing the data was effective in motivating their child to increase their activity (though this was also the case for some buddies). Others felt differently:

"It didn't really make a difference because you can't really change what you do every day.” (100011, child with UCP)

The main perceived benefit of wearing the wristbands that participants with UCP became more aware of their affected arm and seemed to use it more often, as well as being aware that this was something they needed to do in the future. The integration of the approach throughout the day was appreciated, in contrast to formal exercises which were seen as “a bit of a chore”.

"He was getting more involved with things like making his own cereal for his breakfast.” (200001, Mother of child with UCP)

"Towards the end she had this massive, big progress, where she was lifting her own arm because of the Fitbits, so that was a huge improvement than before.” (200010, Buddy, talking about matched participant with UCP)

However, the quality of the movement was a greater concern to some of the therapists.

"But I suppose on my, erm, limited knowledge about what exactly it is measuring would be, is it measuring the quality of the movement? Erm, so we're getting the, the quantity but, you know, how beneficial is that movement to them functionally? And erm, I suppose when I'm thinking about movement, I'm also thinking about, you know, range of movement, function- “ … "spasticity, everything that's acting on those muscles and joints.” (T100001, Physiotherapist)

There was general agreement that the sense of independence provided by the intervention was beneficial:

"Ultimately, children and young people are striving for independence, and this is a tool that has the potential to get them there.” (T200001, OT)

### 3.3. Quantitative data analysis

The five participants who discontinued involvement in the study all did so within the first 3 weeks, and each had very few days with evidence of wristband wear for at least 360 min (all 6 days or less). Their data was not included in the analysis. Of the 33 participants who continued to the end of the study, the mean number of days in which the wristbands were deemed to have been worn for at least 360 min was 63.8 (std 18.1). After exclusion of days where it was likely that the bands had been worn on the wrong wrists), the mean number of days for analysis per participant was 60.7 (std 18.2).

In summary, the arm activity analysis showed a small increase in activity of both arms in children with UCP in the hour just after a prompt compared with the preceding hour, but there was no evidence of a significant increase in either the ratio of arm movement or the absolute movement of the affected arm in the intervention period compared with baseline. The details of this analysis are provided below.

#### 3.3.1. Arm activity analysis

Table 2 provides the summary statistics for arm activity at baseline and in the intervention period. Comparison of the mean non-dominant (affected) hand activity data at baseline vs. the intervention period for participants with UCP using paired t-tests showed no significant difference (*p* = 0.56).

**Table 2:** Summary statistics of arm activity corrected for time worn (minutes) in the baseline and intervention periods.

	Baseline	Intervention
Mean (stdev) activity, dominant arm, UCP	367.2 (97.4)	361.1 (95.9)
Mean (stdev) activity, nondominant (affected) arm, UCP	287.4 (97.1)	281.7 (80.5)
Mean (stdev) activity of dominant arm, Buddy	354.5 (95.4)	367.8 (84.7)
Mean (stdev) activity of nondominant arm, Buddy	340.5 (86.0)	356.8 (82.4)

**Figure 3:**
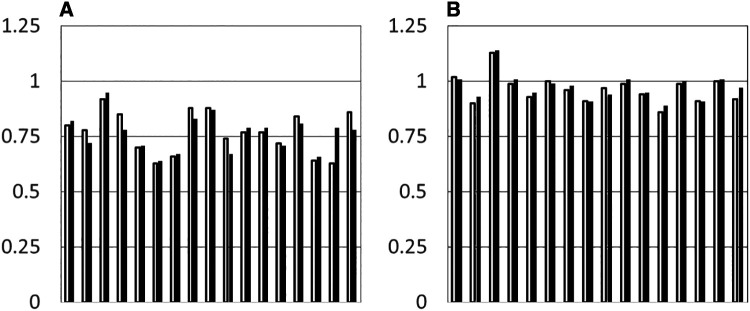
Median ratio of arm movement (non-dominant/dominant) at baseline (grey hars) and in the intervention period (black bars) for each individual participant. (**A**) Participants with UCP. (**B**) Buddies.

Figure 3 shows the median of the daily arm movement ratios at baseline and during the intervention for all participants. In general, participants with UCP had a lower ratio of non-dominant to dominant arm activity than controls, as anticipated, though most controls also moved their non-dominant arm less than the dominant one. The figure shows one clear outlier amongst the buddies, who identified as left-handed but clearly moved his right arm significantly more than his left (arm ratios 1.13 at baseline and 1.14 in the follow up period). The only other left-handed buddy had an arm movement ratio of 1.0 at baseline. There was also an outlier in the participants with UCP (10,003), who had a baseline ratio of arm movement well within the range seen in the buddies (0.92), which increased to 0.95 in the intervention phase. This participant was very mildly affected and indeed had a baseline AHA score of 100 logit units which is the maximum possible score, in keeping with these findings.

The data was then examined for evidence of change in the daily activity ratios over time. The percentage of data points in the intervention period exceeding the median for the baseline period (PEM) was calculated for each participant, where each “data point” was the daily activity ratio (non-dominant/dominant hand).

9/17 children with UCP and 10/16 buddies had a PEM of >50%, which would be a result expected by chance. Only one participant with UCP had a significant TauAvsB value (0.3722, *p* = 0.007), though this was also noted in two buddies. Interestingly the participant with UCP (10,003) had a ratio of arm movement at baseline comparable with that of the group of buddies. This was a 9-year-old, highly motivated child with a very mild hemiparesis. PEM for this child was 71.2%. One other participant with UCP (20,017) showed a clear increase in relative use of the affected arm from baseline to the intervention period, from 63% to 79%, with PEM of 94.7%. However, this participant only had two days of usable baseline data and therefore Tau could not be meaningfully calculated. No participants had significant trendB, therefore Tau AvsB + TrendB was not reported.

#### 3.3.2. Analysis of prompts

The mean number of prompts per person per day (for participants with UCP during the intervention phase) was 5.3 (stdev 1.4) but ranged from 0 to 12. Six participants kept their prompt threshold constant throughout the study. Seven participants decreased their threshold once. One participant decreased their threshold twice. Two participants decreased their threshold three times, one of which was a threshold decrease of 20%. Two participants asked for their decreased thresholds to be just on the weekends. One participant increased their threshold twice. Visual analysis of the number of prompts over time during the intervention period suggested that the change in threshold did not have a clear impact on the number of prompts. Inspection and individual Wilcoxon signed rank tests indicated that there was no significant difference between the number of prompts in the first and second half of the intervention period, except for one participant who had more prompts in the second half after requesting an increase in prompts (decrease in threshold).

Activity in the 5 min after a prompt compared with the 5 min prior to the prompt increased significantly in the non-dominant (affected) arm for 6/17 participants; the same 6 participants also showed significantly increased activity in the dominant arm over the same timescale. Mean activity in the hour after a prompt compared with the hour prior to the prompt was positive in all cases and increased significantly in the non-dominant hand for 10/17 participants; the same 10 participants also showed significantly increased activity in the dominant arm over the same timescale.

The mean difference in activity in the hour after a prompt compared with the hour before was calculated for each participant and converted to a z score to correct for overall differences in activity between participants. The mean of these z scores was 0.261 for the non-dominant hand and 0.247 for the dominant hand indicating a small positive effect size of the prompts.

## 4. Discussion

The first aim of our study was to determine the acceptability of the technology used in different environments and the feasibility of the protocol in terms of commitment for the 10-week period. Our proof-of-concept study demonstrated a general willingness of participants and their families to engage with wrist-worn devices and smartphone applications with the aim of increasing activity of the affected upper limb in UCP. The appeal was perhaps greatest to younger children than teenagers, though some parents of younger children had reservations about their children having access to smartphones. Perhaps installing the application on a tablet and setting strict controls on use would reassure parents; however, this is an area which merits further “patient and public involvement” discussion. Schools and therapists were also generally in support of the approach. However, wear time was less than anticipated. A cut-off of 360 min/day was chosen for analysis, which was only half of the intended daily duration: clearly compliance with a 12 h/day wear time (8 am to 8 pm) over the 10-week period was an unrealistic expectation, with some children requiring later start and/or earlier finish times, and with disruption to wear during the day due to specific activities, weekends and holidays.

The study also highlighted technical challenges which would need to be addressed prior to more widespread testing, in line with our second aim. Technical modifications regarding the process of charging and synchronising the wristbands with the application, improved wristband design tailored to use by children (including waterproof bands useable whilst swimming), and alterations to the game would likely further enhance acceptability. However, other studies using gaming technology for intervention for the upper limb have also struggled with participant compliance (31). Competition with the level of interest and complexity available in commercial games (because of the need to tailor to the patient group and because of constraints on time investment in gaming components in a research setting) is one likely reason (31). To increase reach of the intervention, the smartphone application would need to be compatible with Apple as well as Android devices. Further improvements for ease of use by children with UCP could include use of some form of smartphone holder to facilitate engagement with the application which could otherwise be limited by difficulty holding the phone with the affected hand.

Our final aim was to demonstrate proof of concept that the approach can increase activity of the affected arm in children with UCP. Whilst there was a small increase in arm activity (bilaterally) in the hour after a prompt, there was no overall sustained increase (comparing baseline and intervention periods) in use of the affected arm in children with UCP in our proof-of-concept study. The long-term aim of the approach was to increase activity of the affected arm through everyday activities: it may be that a structured therapy program is required in conjunction with the system for sustained benefit. The approach may be more suited to some children than others. More data would be required to evaluate efficacy. Likewise, one can only speculate on why there was no increase in arm activity in the 5 min after a prompt on average. It is possible that participants felt uncomfortable exhibiting a direct response to the prompt in some settings e.g., a school classroom, or indeed that it might not have been appropriate to increase arm movement at the time.

There is some evidence from the broader literature that the approach used can be effective. For example, Da Silva et al. (32) used similar methodology to ours, with wrist worn devices and prompts, in a cohort of adults following stroke. However, they also incorporated twice-weekly therapy sessions into the intervention. Their study demonstrated that this approach is feasible, though as it was a pilot study, no definitive conclusion regarding benefit can be made. The advantage of combining the system with therapist input is that children could be given a plan for “what to do” on receiving a prompt, which would tie in with their overall therapeutic goals. This still leaves the problem that children may receive prompts at times when it is not appropriate to act on them, for example whilst in a lesson at school. It would be possible to program the devices to accommodate such schedules, avoiding this problem. However, the original problem of lack of therapist time and resources remains. The time commitment involved from the clinical research team in supporting the children and families through this study, troubleshooting, undertaking weekly phone calls should also be acknowledged. Some of the steps required by the research team could be automated in future versions, e.g., altering the threshold required to receive a prompt based on the previous week's arm activity if there had been an increase in arm use. In this study, such steps were undertaken manually.

A particular strength of the study was the use of a buddy system whereby typically developing peers took part in a modified version of the approach, thus providing additional users to test the system but more importantly providing peer support to their buddies with UCP. Another strength was the preceding robust participatory design process with stakeholders (Brown et al., accepted). The ability to undertake remote monitoring of arm activity in a controlled fashion and to allow children with UCP to take ownership of an aspect of their therapy are further strengths. One limitation of the study was the challenge of obtaining detailed feedback from children and young people in the final interviews. Most of the feedback was from parents. The necessity of undertaking interviews online due to the pandemic did not help this situation. With face to face meetings, specific techniques to help elicit children's views of technology could be used in future (33). Another limitation was that children with significant visual impairment, cognitive or language difficulties were excluded as it was felt they would struggle to use the smartphone application.

The COVID-19 pandemic also affected other aspects of this study. Baseline assessments had to be modified to be done virtually. This precluded collection of AHA data for most participants. It is likely that the baseline ratio of affected to unaffected arm activity was a good proxy for this (34), and the ABILHAND-Kids and MACS data provided an adequate summary of baseline function. The Tyneside Pegboard Test was also planned for use whilst wristbands were worn (during the baseline assessment) but this would also have required face to face assessment. Such data, collected from standardised assessments whilst wristbands were worn and videos undertaken, would have started to address another caveat of this type of study which is that increased “activity” is a very nonspecific finding. The nature of the activity is important: increased quality of movements might not equate to increased arm activity, and increased arm activity could in fact represent abnormal movements such as seizures or movement disorder. Whilst it has been known for some time that movement quality is not reflected in accelerometry measures (35), it is still possible that increased arm activity could lead to increased quality of arm movements through the process of practice. Whilst direct evidence for this is lacking, a recent Cochrane review concluded that mechanically assisted walk training slightly improved walking speed and gross motor function in children with cerebral palsy when compared with no walking, but when compared with the same amount of overground walking there was little difference (36).

Complex machine learning-based analysis could begin to unpick these issues, whereas we were limited to an understanding of whether activity of the affected arm could be increased to a higher proportion of that seen in the unaffected arm, as a crude proxy for function in everyday life. Data from the accelerometers matched to a videoed, recorded task, would have given richer information on which to base understanding of the accelerometry data.

The pandemic also disrupted usual routines for school, many sporting activities and therapy appointments for children with UCP. Parents had commented on the adverse impact of these factors on their children. An intervention relying on augmenting a child's everyday activities might not work at its best when those activities were being curtailed due to external pressures.

## 5. Conclusion

This study has shown that use of wrist worn devices and a smartphone application aiming to increase use of the affected arm can be accepted into daily life of individuals with UCP and their families. However, modifications to improve technological issues are required, and whilst there seemed to be a short-term response to prompts, the approach did not lead to sustained increase in activity of the affected arm. Incorporation of a therapy program and increased oversight of the approach would likely be needed to achieve sustained benefit. The use of a buddy system was found to be motivating for some participants and should be explored further in future studies. Ultimately, this technology could improve outcomes for children with UCP due to the possibility of more efficient and less invasive interventions, as well as the ability to assess improvements and give advice remotely.

## Data Availability

The datasets presented in this article are not readily available because The datasets generated for this study are not publicly available in the interests of patient confidentiality. Requests to access the datasets should be directed to anna.basu@newcastle.ac.uk.
